# Comparison of the effect of pharmacotherapy and neuro-feedback therapy on oral health of children with attention deficit hyperactivity disorder

**DOI:** 10.4317/jced.54586

**Published:** 2018-04-01

**Authors:** Ali Vafaei, Iman Vafaei, Gholamreza Noorazar, Rafegh Akbarzadeh, Leila Erfanparast, Sajjad Shirazi

**Affiliations:** 1 Assistant Professor, Department of Pediatric Dentistry, Faculty of Dentistry, Tabriz University of Medical Sciences, Tabriz, Iran; 2 Postgraduate student, Department of Pediatrics, Faculty of Medicine, Tabriz University of Medical Sciences, Tabriz, Iran; 3 Research Center of Psychiatry and Behavioral Sciences, Tabriz University of Medical Sciences, Tabriz, Iran; 4 Student, Faculty of Dentistry, Tabriz University of Medical Sciences, Tabriz, Iran; 5 Associate Professor, Department of Pediatric Dentistry, Faculty of Dentistry, Tabriz University of Medical Sciences, Tabriz, Iran; 6 Lecturer and Researcher, Dental and Periodontal Research Center, Tabriz University of Medical Sciences, Tabriz, Iran

## Abstract

**Background:**

Attention deficit hyperactivity disorder (ADHD) is a chronic and progressive mental disorder related to the childhood period. This study aims to compare the oral health of two groups of ADHD children: those under pharmacotherapy and those under neuro-feedback therapy.

**Material and Methods:**

72 ADHD children (aged 6–12) were divided into two 36 member groups: The pharmacotherapy group and the neuro-feedback therapy group. Unstimulated salivary flow (USF), DMFT, and plaque index were assessed in these children. Statistical analysis was carried out on the data with the independent t-test, which was performed using SPSS 16. The significance level of the study was *p*<0.05.

**Results:**

In this study, the USF of ADHD children who used Ritalin was found to be significantly less than that of the neuro-feedback group (1.25 ± 1.21 vs. 1.91 ± 1.16 ml/min; *p*=0.002). Also, the plaque index (5.9 ± 3.1 vs. 3.94 ± 1.9; *p*=0.018) and DMFT scores (39% ± 9% vs. 31% ± 9%; *p*=0.018) were significantly higher for the pharmacotherapy group.

**Conclusions:**

Neuro-feedback therapy is preferable to Ritalin treatment for ADHD children in terms of their oral health status.

** Key words:**Attention deficit hyperactivity disorder, drug therapy, neurofeedback, oral health.

## Introduction

Attention deficit hyperactivity disorder (AHDH) is a chronic and progressive mental disorder related to the childhood period. An evident characteristic is the manifestation of actions unrelated to the age of the individual such as unrest, doing things or acting without taking situations into account, weak organizational behaviors, and the inability to concentrate. Around 3–7% of school-aged children are affected by this disorder (1,2). Environmental and genetic factors are involved in the accession of ADHD, which leads to a disruption in the operation of dopamine which is a neurotransmitter that influences behaviors like risk-taking and irritability (3).

Treatment of ADHD generally includes combining pharmacotherapy and behavioral-cognitive methods. Although the frontline of the therapy consists of chemical medicines, nowadays non-medicinal methods are favored more due to the side effects caused by drugs, parents’ negative attitude toward medicines, and the probability of the illness recurring after medicine consumption is terminated (4-6). The following are the most prominent non-medicinal methods: Acknowledging and rewarding appropriate behaviors, proper framing of the child’s mind, cognitive-perceptual interventions like neuro-feedback, yoga, meditation etc. Among these, the neuro-feedback therapy method has shown a higher success rate in treating ADHD (7). Recent studies have indicated that combined therapies (behavioral-therapy and pharmacotherapy) may eliminate the need for high drug doses (8).

The frontline of pharmacotherapy for ADHD consists of Methylphenidate (Ritalin) and Dextroamphetamine. Other drugs used to treat this disorder include Bupropion, Risperidone, and tri-cyclic antidepressants (TCA)(9-11). Ritalin is an indirect agonist for dopamine that operates by preventing dopamine from recapturing (1), and is known to have many side effects such as xerostomia and reduced secretion of saliva which lead to extensive dental caries. However, no consensus has been achieved on this issue. Friedlander AH and Friedlander IK showed that pharmacotherapy by Ritalin is not effective on saliva flow (12). On the other hand, Hidas *et al.* found a significant difference in saliva flow, plaque index, and dental caries among ADHD children/adults under Ritalin treatment and those who were not under pharmacotherapy. Meanwhile, the saliva flow was generally lower in ADHD children as compared to normal subjects (13,14).

Considering that no consensus has been attained in the above mentioned studies about the oral side effects of Ritalin in ADHD children, the following question arises: “Are reduced saliva flow and oral side effects the result of the illness itself or due to the pharmacotherapy”? Therefore, this paper investigated oral health status (as indicated by DMFT, plaque index, and unstimulated saliva flow) in ADHD children under pharmacotherapy (Ritalin) and ADHD children under non-medicinal therapeutic methods (neuro-feedback therapy).

## Material and Methods

This cross-sectional/comparative study was performed during September 2015–April 2017 on ADHD children, aged 6–12 years, who were referred to the professional psychiatric clinics of Tabriz University of Medical Sciences. The study protocol was explained fully to the parents/legal guardians, and written informed consents were obtained. The study design was in accordance with the Helsinki Declaration on Ethical Principles for Medical Research Involving Human Subjects and was independently reviewed and approved by the Committee for Research Ethics at Tabriz University of Medical Sciences (Ref number: TBZMED.REC.1394.1076).

Considering α=0.05, power = 80% (15) and 10% difference in unstimulated saliva flow between children under treatment with Ritalin and neuro-feedback therapy (13), a required sample size of 32 was calculated for each group. ADHD in children attending psychiatric clinics was identified by a child and adolescent psychiatrist as per DSM-IV-TR and was approved by K-SADS. The K-SADS is a semi-structured diagnostic interview for children and adolescents aged 6 to 18 years, based on the DSM-IV diagnostic criteria (16). Of the visited children diagnosed with ADHD, 36 children who were only under Ritalin treatment for six months were randomly selected as Group 1. Then, 36 other children who were under only neuro-feedback therapy according to the Linden M. study (17) (40 sessions of 45 minutes each of neuro-feedback therapy for six months) were randomly classified as Group 2. The ADHD children who were under no treatment or used another medicine (other than Ritalin) and those who had systemic or metabolic special diseases were excluded from the study.

-Assessment of Unstimulated saliva flow 

Total unstimulated saliva flow was analyzed in accordance with Aframian *et al.* (accumulating the saliva in the mouth for five minutes and spitting in a glass) (18) and classified as low, very low, and normal according to Ericson and Hardwick (19). Owing to the possible effects of environmental conditions and the time of sampling on the saliva flow (circadian rhythm) samples were taken from all the participants under the same conditions, i.e., between 8 and 12 a.m.in a calm room (20,21). Also, the participants were prevented from eating, drinking, brushing their teeth, or rinsing their mouth for at least one hour before collecting their saliva.

-Assessment of DMFT/dmft and plaque indices in the two groups

DMFT/dmft index was determined by a common method—using a mouth mirror and dental explorer under ample light (22). Plaque accumulation was analyzed by the O’Leary plaque index (23).

-Statistical analysis

The variables were described using mean and standard deviation (SD) and were evaluated by the Kolmogorov-Smirnov test and Q-Q plot to check the normal distribution of data. Levene’s test was used to assess the equality of variances (24,25). The data obtained from the study variables was statistically analyzed with independent t-test using the SPSS16 software (26). *P*<0.05 was considered significant in this study.

## Results

A total of 18 girls and 52 boys with a mean age of 9.8 ± 3.1 years were included into the study.

**Figure 1:**
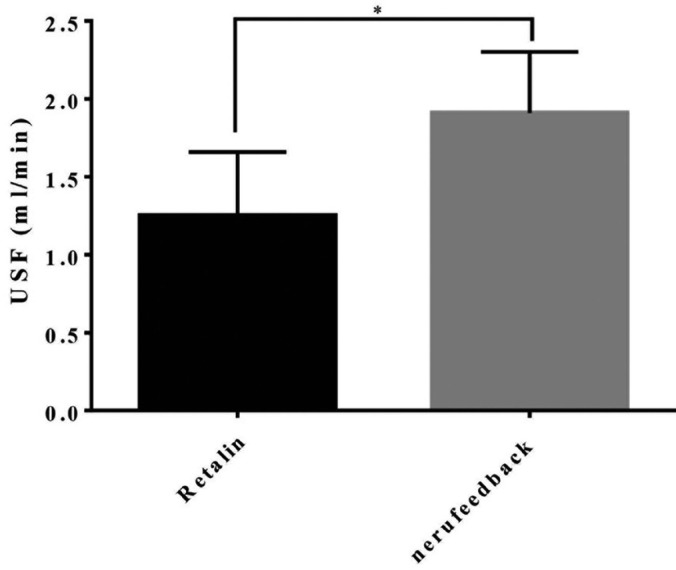
Mean and standard deviation of USF in ADHD children in the study groups.

The mean and standard deviation of USF in ADHD children under Ritalin treatment was 1.25 ± 1.21 ml/min. This figure was 1.91 ± 1.16 ml/min in children receiving neuro-feedback therapy (Fig. 1,  Table 1). The results of the independent t-test showed that the USF of the ADHD children under Ritalin treatment was significantly less than the other group (*p*=0.002).

**Table 1:**
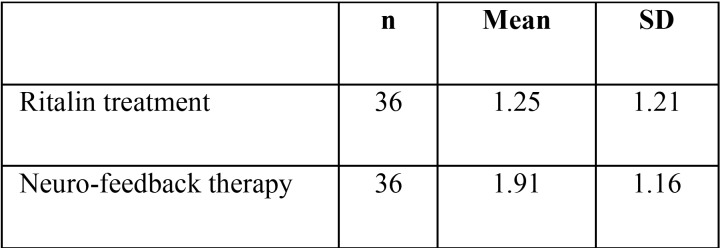
Mean and standard deviation of USF in ADHD children in the study groups.

**Figure 2:**
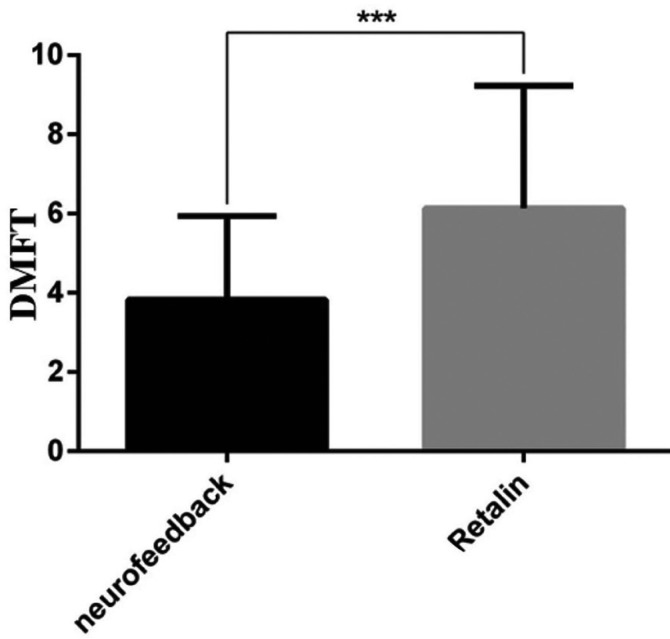
Mean and standard deviation of DMFT scores in ADHD children in the study groups.

The mean and standard deviation of DMFT/dmft scores in ADHD children under Ritalin treatment was 5.9 ± 3.1. This figure was 3.94 ± 1.9 in children receiving neuro-feedback therapy (Fig. 2,  Table 2). The results of the independent t-test showed that the DMFT/dmft scores of the ADHD children under Ritalin treatment was significantly higher than the neuro-feedback therapy group (*p*=0.008).

**Table 2:**
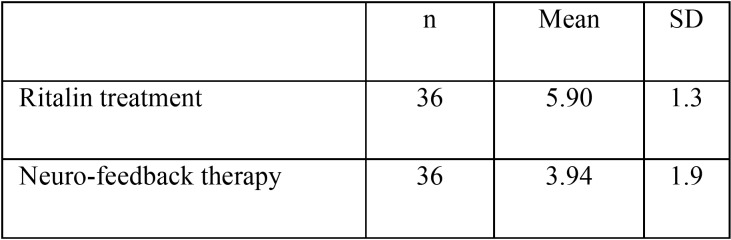
Mean and standard deviation of DMFT scores in ADHD children in the study groups.

**Figure 3:**
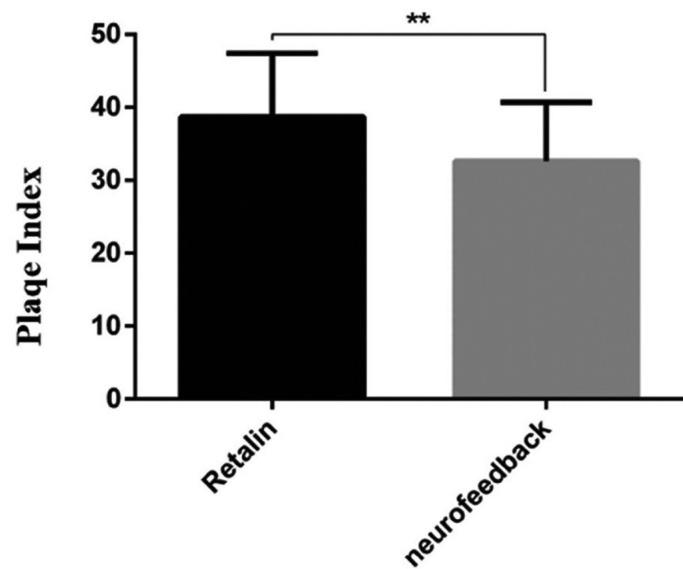
Mean and standard deviation of plaque index scores in ADHD children in the study groups.

 Table 3 and Figure 3 show he mean and standard deviation of the plaque index for both groups. The results of the independent t-test showed that the ADHD children under Ritalin treatment have significantly higher plaque index compared to those under neuro-feedback therapy (39% ± 9% vs. 31% ± 9%, *p*=0.018).

**Table 3:**
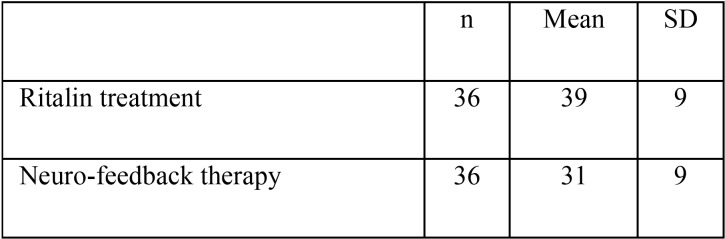
Mean and standard deviation of plaque index scores in ADHD children in the study groups.

## Discussion

This study aimed to compare the oral health of ADHD children under pharmacotherapy and neuro-feedback therapy. The initial symptoms of ADHD usually appear during the preschool/early elementary school years and rarely later than early adolescence (22). ADHD conveys a risk for the development of dental anxiety, depressive and anxiety disorders as well as learning impairments, mood and language disorders (22,27). These conditions make the dental treatment time-consuming, costly and demanding for the clinician and the child, and have a strong negative impact on treatment outcome (27,28). ADHD children possess coping problems in challenging situations like dental setting owing to their impaired cognitive condition and higher rate of learning disorders (22,29). This highlights the importance of caries prevention and selection of the treatment modality which does not compromise the oral health of ADHD children. Our results showed that USF in the ADHD children who were treated with Ritalin was significantly lower compared to those who were treated with neuro-feedback therapy. In addition, the plaque index and dental caries were significantly higher in children receiving Ritalin.

Although there is some inconsistency in the literature, most studies have revealed unhealthy oral status and higher caries among ADHD children (22,30,31). Four theories have been proposed with respect to the occurrence of higher caries in children with ADHD. The nature of the illness itself results in poor oral hygiene; a cariogenic diet full of sugar which is consumed a lot by these patients; mouth dryness due to the side effects of medicines; and the lack of attention given by parents toward these children’s oral hygiene (32). In addition, previous research shows that fear, stress, and noncooperation occurs in between 5.7% and 6.7% of children when attending the dentist, and this figure is higher in ADHD children which may prevent them from revisiting the dentist and may be another important reason for their high dental caries (9,10,33).

USF in ADHD children undergoing treatment with Ritalin was significantly lower as compared to those under the neuro-feedback treatment. Ritalin is an indirect agonist for dopamine and operates by preventing the dopamine from recapturing (1). Xerostomia, and reduced secretion of saliva in the dental and oral section, are the most important side-effects of these medicines, which lead to extensive dental caries (13,14,32). A study by Medori *et al.* showed that Ritalin-based oral dryness is dose-dependent, and 8.11% of their patients reported mouth dryness. On the other hand, only 1.2% of subjects in placebo group reported dryness of mouth (18).

In addition, reduced saliva flow in the ADHD children who use drugs may increase caries in these children (7,8). Similarly, drug-using children had higher dental caries in our study. In this line, Aminabadi *et al.* study (22) showed that children with ODD/ADHD had significantly higher DMFT/dmft scores than normal children. Contrary to the results obtained in this paper, a study by Grooms *et al.* (2005) could not show a significant difference in terms of USF in children with ADHD using different medicines as compared to normal children. However, DMFS/dmfs was higher in this group than the control group (34). The reason behind this contradiction is the non-discrimination of children into drug users and non-drug users, which may affect the results. In addition, n a case-control study on children aged 11–13 years, the consumption of drugs was related to a high DMFT in ADHD children. However, this study did not take medicine as a probable risk factor in dental caries. Also, this study was not reliable as it had a small sample size and only nine out of 14 ADHD children were under pharmacotherapy (35).

Furthermore, the age spectrum could be a confounding factor in obtaining contradictory results. For example, in a study by Blomqvist *et al.* (2007) on 13-year-old children with ADHD, no significant difference was observed in terms of DMFT between normal and ADHD subjects (30). Another study by Blomqvist *et al.* (2011) on 17-year-old children showed that ADHD children had a higher DMFT than the control group (31). According to these studies, the underlying factor of caries in the freshly-grown teeth of ADHD children does not have sufficient opportunities to affect them.

Aminabadi *et al.* study (22) showed that there was no significant difference in gingival index between children with ODD/ADHD and normal children. However, we found that the plaque index was higher in ADHD children taking drugs, in addition to high DMFT and low USF which indicates the effects of USF on the accumulation of plaque in the Ritalin-using group. It also may be the more serious effect of neuro-feedback therapy on controlling the behavioral problems of this group of children that improves their hygiene behaviors which requires further studies.

Generally, only two studies by Ariela Hidas (2011 and 2012) analyzed the saliva and oral status of ADHD children. It was done by separating them into subjects under pharmacotherapy and subjects undergoing no treatment (13,14). In their study, although USF was lower in ADHD children than the normal ones, yet no significant difference was observed between two ADHD groups, including those who took medicines and those without any treatment. Also, no significant difference was observed in terms of DMFT between the normal children, ADHD children under Ritalin treatment, and ADHD children not undergoing pharmacotherapy (14,17). An important difference between this study and the study by HIDAS is the higher number of samples and the longer duration of medication, which may affect the results. On the other hand, in this study, both the groups of patients were under pharmacotherapy or neuro-feedback therapy for six months, which may reduce the intervening factor of treatment duration between the two groups.

In overall, our results showed better oral health for ADHD children under neuro-feedback therapy which indicates the importance of therapies for ADHD children that are non-medicinal and less invasive. These types of treatments result in fewer dental and oral side effects in addition to the improvement of behavioral problems in ADHD children. Although the results of this study indicate lower USF and higher PI in ADHD children under pharmacotherapy, because of the multitude of factors influencing dental caries and many intervening factors in this kind of study, further studies are required to make conclusions with certainty and resolve these contradictions, specifically by using a bigger sample size and eliminating other influencing factors.

## Conclusions

The results of this study showed that children with ADHD undergoing medical treatment by Methylphenidate (Ritalin) had lower unstimulated saliva flow (USF) index and higher DMFT and plaque index (PI) than ADHD children under neuro-feedback therapy. Therefore, Neuro-feedback therapy is preferable to Ritalin treatment for ADHD children in terms of their oral health status.
